# Grape Extract Promoted α-MSH-Induced Melanogenesis in B16F10 Melanoma Cells, Which Was Inverse to Resveratrol

**DOI:** 10.3390/molecules26195959

**Published:** 2021-10-01

**Authors:** Siqi Zhou, Drira Riadh, Kazuichi Sakamoto

**Affiliations:** Faculty of Life and Environmental Sciences, University of Tsukuba, Tsukuba 305-8572, Ibaraki, Japan; zhousiqi66@gmail.com (S.Z.); riadh.drira@gmail.com (D.R.)

**Keywords:** melanin synthesis, B16F10 melanoma cells, grape extract, resveratrol, tyrosinase

## Abstract

Melanin is a natural pigment produced by cells to prevent damage caused by ultraviolet radiation. Previously, resveratrol was shown to reduce melanin synthesis. As a natural polyphenol with various biological activities, resveratrol occurs in a variety of beverages and plant foods, such as grapes. Therefore, we investigated whether grape extracts containing resveratrol also had the ability to regulate melanin synthesis. In this study, we used mouse B16F10 melanoma cells as a model for melanin synthesis with the melanogenesis-inducing α-melanocyte-stimulating hormone (α-MSH) as a positive control. Our results confirmed previous reports that resveratrol reduces melanin synthesis by reducing the activity of the rate-limiting enzyme tyrosinase. In contrast, the grape extract could not reduce melanin synthesis, and in fact promoted melanogenesis in the presence of α-MSH. The expression of genes related to melanin synthesis, such as tyrosinase, tyrosinase-related protein-1, tyrosinase-related protein-2, and microphthalmia-associated transcription factor, also supports these phenomena, which means that even in the presence of resveratrol, grape extract will strengthen the function of α-MSH in promoting melanin synthesis. Therefore, these results also provide a point of view for research on cosmetics.

## 1. Introduction

Melanin, is produced by melanocytes in the hair follicles and the epidermis of the skin, and is essential for human skin pigmentation and protection from ultraviolet (UV) radiation damage [1,2,3]. Melanin synthesis is mainly catalyzed by tyrosinases, tyrosinase-related protein-1 (TRP-1), and tyrosinase-related protein-2 (TRP-2), which is also called dopachrome tautomerase [4,5]. Tyrosinase converts tyrosine to L-3,4-dihydroxyphenylalanine (L-DOPA), which then oxidizes to form dopaquinone [6]. Subsequently, TRP-1 and TRP-2 catalyze the conversion of dopaquinone to melanin [7,8]. Notably, tyrosinase, TRP-1, and TRP-2 mainly participate in the synthesis of eumelanin, one of the two types of melanin produced in mammals that have a black/brown color. In contrast, pheomelanin has a red/yellow color. The beneficial effects of melanin are attributed to eumelanin, whereas pheomelanin has been associated with tumorigenesis [9].

Melanin synthesis is induced by α-melanocyte-stimulating hormone (α-MSH), which is secreted by keratinocytes exposed to UV radiation [10]. α-MSH engages the melanocortin-1 receptor and activates cyclic AMP (cAMP) signaling via a G-protein transporter. In turn, cAMP activates protein kinase A, which then induces the expression of CRE-binding protein (CREB) via the cAMP regulatory element (CRE) in its promoter region. Subsequently, CREB induces the expression of microphthalmia transcription factor (MITF), which finally induces the downstream target genes encoding for tyrosinase, TRP-1, and TRP-2, to promote melanin synthesis [11].

Resveratrol (3,5,4′-trihydroxy-trans-stilbene) is a natural polyphenol found in various plants, including grapes, blueberries, and peanuts [12,13]. Research on the “French paradox” phenomenon showed that consumption of red wine and its component resveratrol reduces the risk of cardiovascular disease [14]. Recently, it was discovered that resveratrol also ameliorates cardiovascular damage caused by other diseases [15]. Additionally, a resveratrol-rich diet markedly reduced the incidence and multiplicity of carcinogen-induced mammary tumors in mice [16]. Furthermore, the antioxidant, anti-inflammatory, and anti-aging effects of resveratrol have been described [15,17,18,19]. Recently, the compound was shown to induce depigmentation in human melanocytes and animal models [13,20]. However, the melanogenesis-regulating effect of resveratrol-rich red wine or grape extract has rarely been studied. Therefore, in this study we investigated whether concentrated grape extracts similar to wine (the main raw materials of wine include grape pericarp, flesh, seeds, and stems) can regulate melanin synthesis.

In this study, we confirmed the depigmentation effect of resveratrol, whereas the grape extract yielded a contrasting result.

## 2. Results

### 2.1. Effects of Grape Extract and Resveratrol on Cell Viability

**Figure 1:**
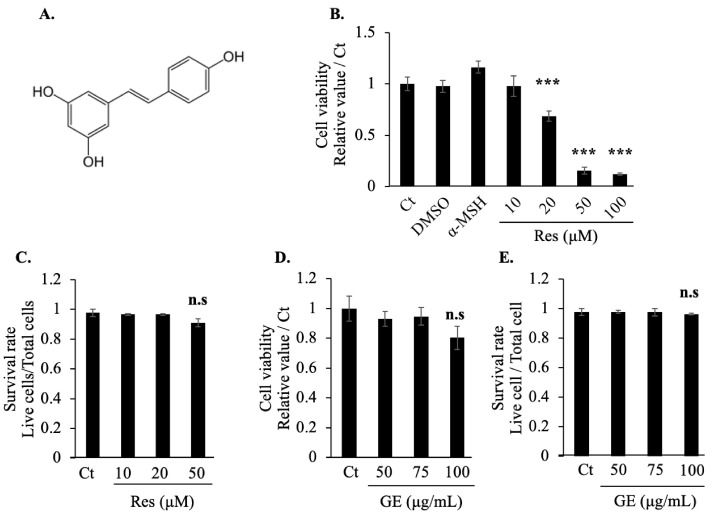
Effect of resveratrol (Res; **A**) and grape extract (GE) on B16F10 melanoma cell viability. (**B**) Cell viability after 72 h of treatment with Res (10, 20, 50, or 100 μM) or 10 nM α-MSH, measured using an 3-(4,5-dimethylthiazol-2-yl)-2,5-diphenyltetrazolium bromide (MTT) assay. (**C**) Cell survival rate (live cell count/total cell count) after 72 h of treatment with Res (10, 20, or 50 μM). Trypan blue staining was used to count the live and dead cells. (**D**) Cell viability and (**E**) survival rate after 72 h of treatment with GE (50, 75, or 100 μg/mL), measured using an MTT assay and trypan blue staining, respectively. The results are presented as the mean ± standard deviation; *n* ≥ 3; *** *p* < 0.001 vs. Ct (non-treatment group). n.s. means non-significant. Dimethyl sulfoxide (DMSO) at a safe concentration (0.1% v/v) was the solvent for Res, GE, and α-MSH.

Resveratrol is a natural polyphenol (Figure 1A). Initially, we tested the cytotoxicity of resveratrol using a 3-(4,5-dimethylthiazol-2-yl)-2,5-diphenyltetrazolium bromide (MTT) assay. Dimethyl sulfoxide (DMSO) was used as the solvent in this experiment; concurrent cytotoxicity testing of DMSO and the positive control for melanin synthesis (10 nM α-MSH) revealed no cytotoxic effects, but high concentrations of resveratrol (50 μM and 100 μM) seemed to have a greater killing effect on cells (Figure 1B). Additional confirmation of the toxicity of resveratrol was obtained using a trypan blue assay, in which no cytotoxic effect was noted, even at the highest tested dose of resveratrol (50 μM) (Figure 1C). Therefore, we selected relatively safe resveratrol concentrations (10 μM and 20 μM) for the subsequent experiments.

We also used the same method to evaluate the cytotoxicity of the grape extract, which caused no cell death even at the highest concentration of 100 μg/mL. Therefore, we used two concentrations of grape extract (50 μg/mL and 100 μg/mL) in the subsequent experiments.

### 2.2. Resveratrol and Grape Extract Have Inverse Effects on α-MSH-Induced Melanogenesis

**Figure 2:**
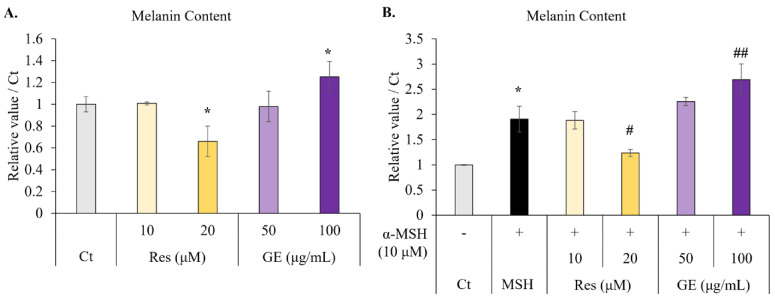
Effect of 72 h treatment with resveratrol (Res; 10 μM or 20 μM) and grape extract (GE; 50 μg/mL or 100 μg/mL) on melanogenesis in B16F10 melanoma cells. The positive control for melanin synthesis was α-melanocyte-stimulating hormone (α-MSH). The absorbance at a wavelength of 405 nm was measured after 72 h. The relative melanin content was normalized to the total protein content of the cell. (**A**) The melanin content in cells without α-MSH stimulation. (**B**) The melanin content in cells stimulated with α-MSH (10 μM). The results are indicated as the mean ± standard deviation, *n* ≥ 3. * *p* < 0.05 vs. Ct (non-treatment), # *p* < 0.05, ## *p* < 0.01 vs. α-MSH.

To verify the ability of resveratrol to reduce melanin synthesis and to test whether grape extract can regulate melanin synthesis, we treated B16F10 cells directly with resveratrol and grape extract, followed by measurement of the intracellular melanin content. Similar to previous studies, resveratrol reduced the melanin content of the melanocytes. Interestingly, the grape extract showed a weak ability (approximately 1.2 times) to promote melanin synthesis (Figure 2A).

We then tested the effects of resveratrol and grape extract on melanin synthesis in the presence of α-MSH. In the positive group, α-MSH significantly increased melanin production. Moreover, we found that resveratrol also reduced melanin content in the presence of α-MSH, while grape extract, in contrast, significantly promoted the synthesis of melanin in the presence of α-MSH (Figure 2B).

### 2.3. Effect of Resveratrol and Grape Extract on Intracellular Tyrosinase Activity

**Figure 3:**
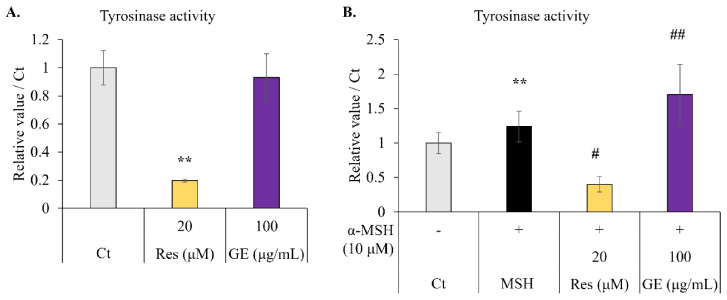
Effect of 72 h treatment with resveratrol (Res; 20 μM) and grape extract (GE; 100 μg/mL) on intracellular tyrosinase activity in B16F10 melanoma cells. The positive control for melanin synthesis was α-melanocyte-stimulating hormone (α-MSH). After 72 h, L-DOPA was added to the cell lysate, and the tyrosinase activity was expressed in terms of the amount of melanin synthesized after 20 min, normalized to the total protein content of the cell. (**A**) Intracellular tyrosinase activity in melanocytes without α-MSH treatment. (**B**) Intracellular tyrosinase activity in melanocytes stimulated with α-MSH (10 µM). The results are expressed as the mean ± standard deviation, *n* ≥ 3, ** *p* < 0.01 vs. Ct (non-treatment); # *p* < 0.05, ## *p* < 0.01 vs. α-MSH.

We tested the effects of resveratrol and grape extracts on the activity of tyrosinase, the key rate-limiting enzyme in melanin synthesis. When B16F10 cells were treated with resveratrol or grape extract alone, resveratrol inhibited tyrosinase activity, but the grape extract had no effect (Figure 3A).

We then tested the effects of resveratrol and grape extract on melanocytes in the presence of α-MSH. The stimulating effect of α-MSH on tyrosinase activity was apparent; treatment with resveratrol (20 µM) inhibited this effect. However, when α-MSH (10 µM) and grape extract (100 µg/mL) were combined, tyrosinase activity was enhanced (Figure 3B). Therefore, we concluded that resveratrol could inhibit tyrosinase activity and grape extract could amplify the effect of α-MSH.

### 2.4. Effect of Resveratrol and Grape Extract on Melanogenesis-Related Gene Expression

**Figure 4:**
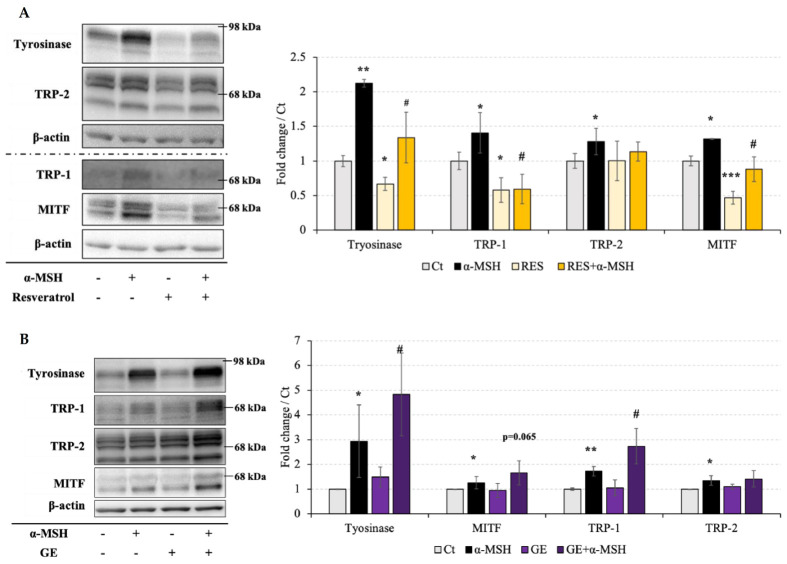
Effect of 72 h treatment with resveratrol (Res; 20 μM) and grape extract (GE; 100 μg/mL) on melanogenesis-related gene expression in B16F10 melanocytes. The positive control was α-melanocyte-stimulating hormone (α-MSH; 10 nM). Western blotting was performed to evaluate the expression of tyrosinase (90 kDa), tyrosinase-related protein-1 (TRP-1) (70 kDa), tyrosinase-related protein-2 (TRP-2) (60–80 kDa), and microphthalmia-associated transcription factor (MITF) (60–80 kDa) following treatment with (**A**) Res and (**B**) GE. The internal control was β-actin. The results are presented as the mean ± standard deviation, *n* ≥ 3, * *p* < 0.05, ** *p* < 0.01, *** *p* < 0.001 vs. Ct (non-treatment); # *p* < 0.05 vs. α-MSH.

In the experiment described in Section 2.3, we investigated the effects of resveratrol and grape extract on tyrosinase activity. We then analyzed the gene expression of melanin-synthesis-related factors. First, we tested the regulatory effect of resveratrol on the expression of tyrosinase, TRP-1, TRP-2, and their transcription factor, MITF. α-MSH was used as a positive control to regulate melanin synthesis. Consistent with our expectations, α-MSH promoted the expression of all the related factors. Resveratrol significantly reduced the expression of tyrosinase, TRP-1, and MITF. However, resveratrol did not significantly alter the expression of TRP-2 (Figure 4A).

Next, we detected the effect of grape extract on the gene expression of melanin-synthesis-related factors. We found that grape extract had no obvious effect on the factors related to melanin synthesis. However, in the presence of α-MSH, grape extract further promoted the expression of tyrosinase/TRP-1 compared to α-MSH (Figure 4B). The transcription factor MITF was also further promoted, but unfortunately there was no significant difference in this promotion effect (*p* = 0.065 vs. α-MSH).

Based on these results, we investigated the ability of resveratrol to reduce the expression of most melanin-synthesis-related factors. However, grape extract expanded the effects of α-MSH on the gene expression of melanin-synthesis-related factors.

## 3. Discussion

Resveratrol has been studied extensively since its discovery; besides its protective effect against cardiovascular disease, many other physiological effects have also been discovered in recent years, such as anti-oxidation, anti-inflammatory, and anti-aging effects [15,17,18,19]. Some recent studies have also reported the effects of resveratrol on the functions of various cell types, such as reduced lipogenesis in 3T3-L1 cells [21] and the promotion of myogenesis in C2C12 cells [22]. 

Resveratrol also has an ameliorative effect on some age-related diseases such as Parkinson’s disease and Alzheimer’s disease [23], in addition to an attenuating effect on cancer cells at various stages [24]. In this study, we used resveratrol to treat B16F10 mouse melanoma cells. Similar to results obtained using other cancer cell lines, our data showed that 50 μM and 100 μM resveratrol treatment significantly inhibited cell activity. However, we found that 50 μM resveratrol did not cause significant cell death, but the number of cells was significantly reduced by treatment with 20 μM and 50 μM resveratrol. Therefore, we tested the effect of 20 μM resveratrol on cell proliferation. Consistent with our hypothesis, resveratrol significantly inhibited cell proliferation compared with the control group (Figure S1A), which is consistent with the results of previous anti-cancer studies. However, in most previous studies, the concentration of resveratrol was not uniform. Some experiments have shown that a high concentration of resveratrol (above 100 μM) is required to inhibit cancer or protect against cardiovascular disease. In previous studies on the melanogenesis-inhibiting effects of resveratrol, some researchers also used 100 μM or higher concentrations. Herein, we found it difficult to maintain cell viability at a concentration of 100 μM [13]. Because the purpose of this experiment was to explore the effect on melanogenesis, relatively safe resveratrol concentrations of 10 μM and 20 μM were selected. However, choosing an appropriate resveratrol concentration in future experiments or clinical scenarios will bring new challenges.

Grape, a plant fruit rich in resveratrol, was selected as the main research object in this study. After the grapes were dried, crushed, extracted with alcohol, dried again, and re-dissolved, we measured the resveratrol content in the samples; 50 μg/mL grape extract contained 15 μM resveratrol. Compared with resveratrol, the grape extract showed lower cytotoxicity and inhibited cell proliferation (Figure S1B). Therefore, we can conclude that grape extract exhibits lower cytotoxicity than resveratrol. However, it is unclear whether this inhibitory effect promotes (via lower toxicity) or inhibits (via weakened anti-cancer effect) the effect of resveratrol.

Next, we compared the effects of resveratrol and grape extract on the regulation of melanin synthesis. Similar to the results of previous studies [13], resveratrol inhibited melanin synthesis, although we used a lower concentration to obtain similar results in this experiment. This effect was verified by the results of additional assays of tyrosinase activity and gene expression of melanogenesis-related factors. In contrast, grape extract containing the same concentration of resveratrol did not significantly regulate melanin synthesis, but in the presence of α-MSH, the grape extract increased the melanin content within the cells. Subsequent tyrosinase activity testing and Western blotting showed that grape extract plus α-MSH also promoted the activity and expression of tyrosinase compared with the positive control group of α-MSH. This shows that the grape extract used in this study had the ability to promote the melanogenesis-stimulating effect of α-MSH, but did not have a significant effect on melanin synthesis by itself.

These results have prompted new questions and future research directions—first, how does grape extract increase the effect of α-MSH without affecting melanin synthesis? α-MSH activates the cAMP pathway by binding to receptors on the cell membrane, leading to the activation of MITF expression and, subsequently, tyrosinase expression, thereby promoting melanogenesis [11,25]. However, more than one pathway regulates melanin synthesis in melanocytes; there are also several factors that regulate the expression and activity of MITF, such as mitogen-activated protein kinases (extracellular-regulated kinase [ERK], p38, and c-Jun N-terminal kinase) [26,27], PI3K/Akt, β-catenin, and glycogen synthase kinase 3β [28,29,30]. Among them, the ERK pathway promotes the degradation of MITF, thereby inhibiting melanin synthesis. We also initially tested this pathway and found that the grape extract had no effect on the phosphorylation (activity) of ERK; however, treatment with grape extract plus α-MSH reduced the phosphorylation of ERK (Figure S2). Therefore, we suspect that the grape extract exerted its effect via the ERK pathway; further analyses and research are required considering the complex metabolic pathways of melanogenesis. Moreover, it is not only the signal pathway related to melanin synthesis but also how GE affects the effect of α-MSH (e.g., the binding ability of α-MSH to the receptor, the expression of MC1R, and the activity of cAMP-PKA-CREB signaling pathway) which can help us to better understand how GE can promote α-MSH-induced melanogenesis.

The second question prompted by our results was why the grape extract and resveratrol had different effects on melanocytes. Many secondary metabolites have been identified in grapes, such as polyphenols, linoleic acid, procyanidins, phenolic acids, and flavonoids [31]. Linoleic acid exhibits an inhibitory effect on melanogenesis [32]. Research on gallic acid (phenolic acid) [33] and catechin/epicatechin (flavonoids) [34] suggested that these compounds downregulate skin cell pigmentation. Quercetin, one of the main flavonoids, showed inconsistent results, dependent on cell type and species, in melanogenesis assays [35]. Furthermore, natural procyanidins and anthocyanins found in blueberries [36] have been shown to promote melanogenesis in human pigment cells [35,37]. We also found a recent paper that reported that an extract of Neo Muscat grapes inhibited the synthesis of melanin [38], contrary to our results. We suspect that the discrepancy may be attributed to the different colors of the grapes (i.e., different anthocyanin composition), and anthocyanins may be the major component in grape extract that inhibits the effect of resveratrol. Our future aim is to conduct a component analysis of the grape extract to explain why its effect on melanogenesis differs from that of resveratrol.

## 4. Materials and Methods

### 4.1. Materials

B16F10 melanoma cells were obtained from RIKEN BRC (Tsukuba, Japan). RPMI-1640 medium, MTT, and bovine serum albumin (BSA) were purchased from Sigma-Aldrich (Tokyo, Japan). Trypan blue and 2′,7′-dichlorofluorescein diacetate powder were purchased from Wako (Osaka, Japan) and Cayman Chemical (Ann Arbor, MI, USA), respectively. Cell culture dishes (10 cm), 6-well, 24-well, and 96-well plates were purchased from TPP (Trasadingen, Switzerland).

### 4.2. Grape Extract Sample Preparation

Crushed grape powder (including pericarp, seed, flesh, and grape stem) (Zero Co. Ltd., Chiba, Japan) was dissolved in 70% ethanol, macerated for 7 days, and centrifuged at 3000 rpm for 20 min. The supernatant was filtered through a 0.22 μm membrane and completely dried. The resulting grape extract was dissolved in DMSO and stored at –80 °C. Resveratrol (Wako, Osaka, Japan) was also dissolved in DMSO at 100 mM and stored at −80 °C. A UPLC system (UPLC–SYNAPT G2-S HDMS) from Waters Co. (MA, USA) was used to roughly calculate the resveratrol content in the grape extract (Figure S3). HPLC separation was achieved on a 2.1 × 150 mm column (Waters Co., MA, USA). The mobile phases consisted of (A) water and (B) methanol. The separation was carried out at room temperature with a 0.2 mL/min flow rate, under the following conditions: 0–30 min, 0–50% B, 30–50 min, 50–95 % B, 50–55 min, 95 % B isocratic, 55–60 min, 100–0% B, followed by a 5 min re-equilibration time of the column. Calculated by comparing the peak area at the same position, the grape extract contained 7% resveratrol, so the concentration of resveratrol in 50 μg/mL (100 μg/ml) grape extract was determined to be about 15 μM (30 μM).

### 4.3. Cell Culture

B16F10 melanoma cells were cultured at 37 °C and 5% CO_2_ in RPMI-1640 medium containing 10% fetal bovine serum (HyClone, Logan, UT, USA). Before reaching confluence, the cells were split into a 6-well plate (3.4 × 10^4^/well) or 10 mm dish (2.5 × 10^5^/dish), allowed to adhere for 24 h before treatment with resveratrol, grape extract, or α-MSH, as described in Section 4.4 and 4.5.

### 4.4. MTT and Trypan Blue Assay

For the MTT assay, B16F10 cells were seeded in a 24-well plate at 3.0 × 10^3^ cells/well. After exposure for 72 h to grape extract (50, 75, or 100 μg/mL) or resveratrol (10, 20, 50, or 100 μM), the media were replaced with fresh media supplemented with 500 μg/mL MTT. The cells were then incubated at 37 °C for 4 h and treated with isopropanol in 0.04 M HCl to dissolve purple formazan crystals that may have formed. Absorbance values at 570 nm and 630 nm were then measured using a microplate reader. For the trypan blue assay, the cells were plated in a 6-well plate at 3.4 × 10^4^ cells/well and exposed to grape extract (50, 75, or 100 μg/mL) or resveratrol (10, 20, or 50 μM) for 72 h. The cells (including any that were floating in the medium) were then collected by trypsinization and resuspended in phosphate-buffered saline containing 0.5% trypan blue. The stained cells were counted using a cell counter.

### 4.5. Melanin Content

B16F10 melanoma cells were seeded at 3.4 × 10^4^ cells/well in a 6-well plate. After 72 h of treatment with grape extract (50 μg/mL or 100 μg/mL) or resveratrol (10 μM or 20 μM; 10 nM of α-MSH was used as a positive control for melanin synthesis), the cells were collected by trypsinization and suspended in 150 μL of 1 N NaOH. After heating at 80 °C for 1 h, the absorbance at 405 nm was measured using a microplate reader. The final relative melanin content was normalized to the total protein content, measured using a BCA assay kit (Thermo Fisher Scientific K.K., Tokyo, Japan).

### 4.6. Intracellular Tyrosinase Activity

B16F10 melanoma cells were seeded at 3.4 × 10^4^ cells/well in a 6-well-plate, treated with or without 10 nM α-MSH, grape extract (100 μg/mL), or resveratrol (20 μM) for 72 h, harvested by trypsinization, resuspended in phosphate buffer containing nonionic surfactant (10% Triton X-100), sonicated, and centrifuged at 14,000 rpm for 20 min. The supernatant was collected and assayed for total protein content; 50 μg was mixed with 2 μL of 10% (w/v) L-DOPA (Sigma Aldrich, Tokyo, Japan) in phosphate buffer. Subsequently, absorbance at 475 nm was measured using a microplate reader.

### 4.7. Western Blot

B16F10 melanoma cells were seeded at 2.5 × 10^5^ cells/well in a 10 cm dish. After treatment with grape extract (100 μg/mL) or resveratrol (20 μM) for 72 h, cells were collected in radioimmunoprecipitation assay buffer (50 mM Tris-HCl (pH 7.4), 150 mM NaCl, 1 % Triton X-100, 0.1 % sodium dodecyl sulfate (SDS), 0.5 % deoxycholate-Na, 1 mM ethylenediaminetetraacetic acid, 10 mM NaF, and protease inhibitor), sonicated, and centrifuged at 14,000 rpm for 20 min. The resulting supernatant was collected, and 20 μg of protein from each sample was separated by SDS-polyacrylamide gel electrophoresis and transferred to a polyvinylidene difluoride membrane. The membrane was then blocked for 1 h with 2% BSA and probed overnight at 4 °C with primary antibodies against MITF (Cell Signaling Technology, Danvers, MA, USA), tyrosinase, TRP-1, and TRP-2 (Santa Cruz Biotechnology, Dallas, TX, USA). Subsequently, the membranes were probed for 60 min at room temperature with the appropriate secondary antibodies, stained with LuminoGLO (Cell Signaling Technology), and visualized using an AE-9300H Ez-Capture MG imaging system (ATTO Corporation, Tokyo, Japan).

### 4.8. Statistical Analysis

The statistical significance of all data was determined using Student’s *t*-test. The Western blot data were analyzed using ImageJ (https://imagej.nih.gov/ij/; accessed on 13 April 2020). Statistical significance was set at *p* < 0.05.

## 5. Conclusions

Resveratrol inhibits melanogenesis by suppressing the expression of melanogenic proteins. In contrast, the grape extract had no effect on melanin synthesis, but enhanced α-MSH-induced melanin synthesis.

## Data Availability

Data are contained within the article or supplementary material.

## Supplementary Materials

The following are available online. Figure S1: The proliferation of B16F10 melanoma cells exposed to resveratrol or grape extract. Figure S2: Grape extract decreased the relative phosphorylation level of ERK. Figure S3: HPLC analysis for resveratrol and grape extract.

## Sample Availability

The samples used in this study are rare and cannot be provided but can be purchased commercially.
