# Supplementary figures and images for: Grape Extract Promoted α-MSH-Induced Melanogenesis in B16F10 Melanoma Cells, Which Was Inverse to Resveratrol
Source: Molecules. 2021 Oct 1;26(19):5959. doi: 10.3390/molecules26195959 (PMC8512250; doi:10.3390/molecules26195959)

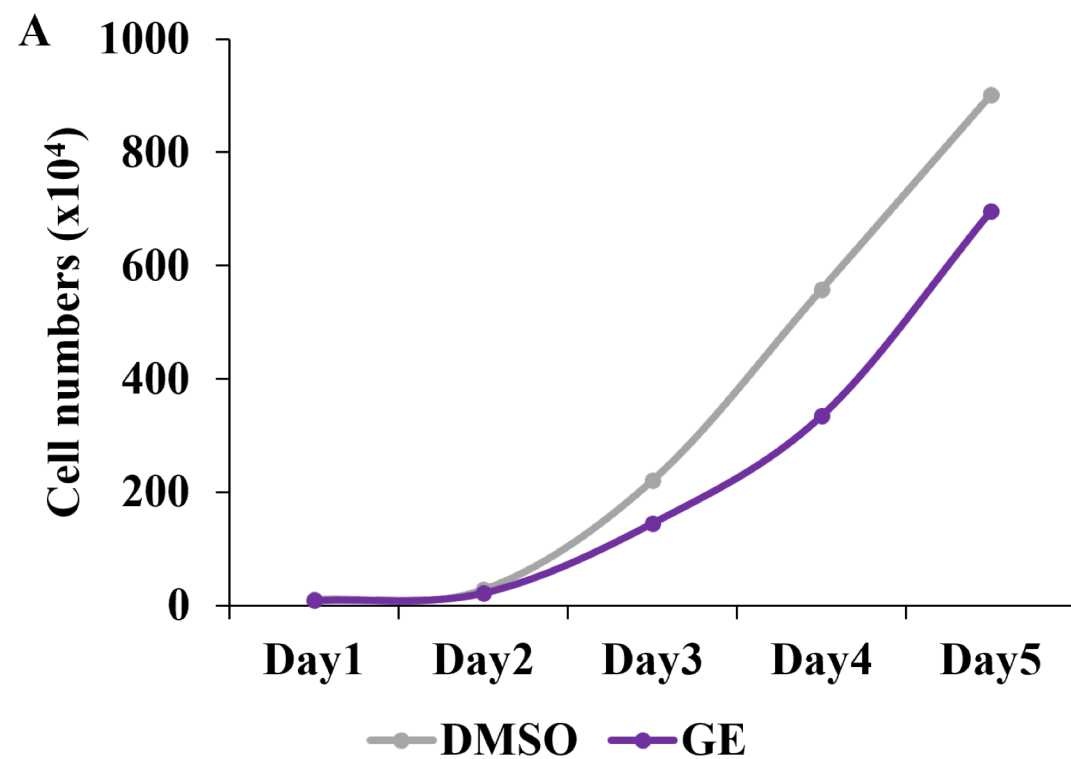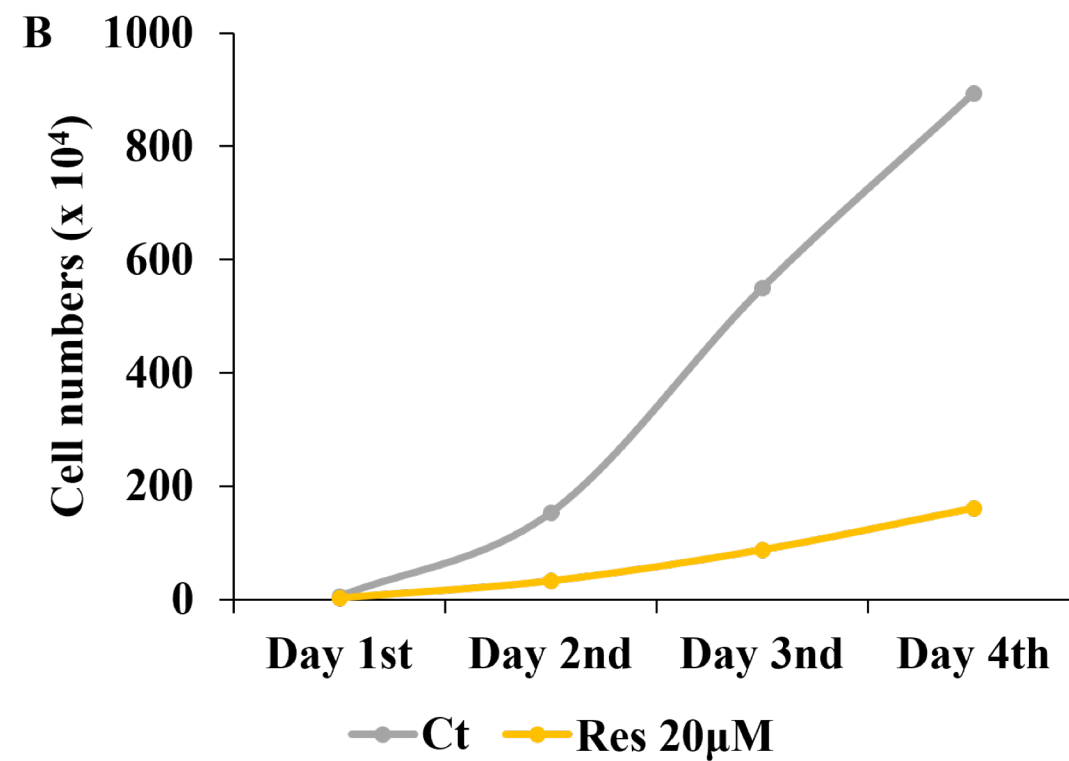

**Figure S1:** The proliferation of B16F10 under the treatment of resveratrol or grape extract.

Supplement: Supplementary file 1 [file molecules-26-05959-s001.zip › Molecules-Supplemental FigureS1.pdf]

27-Jan-2016 23:53:39  
Res

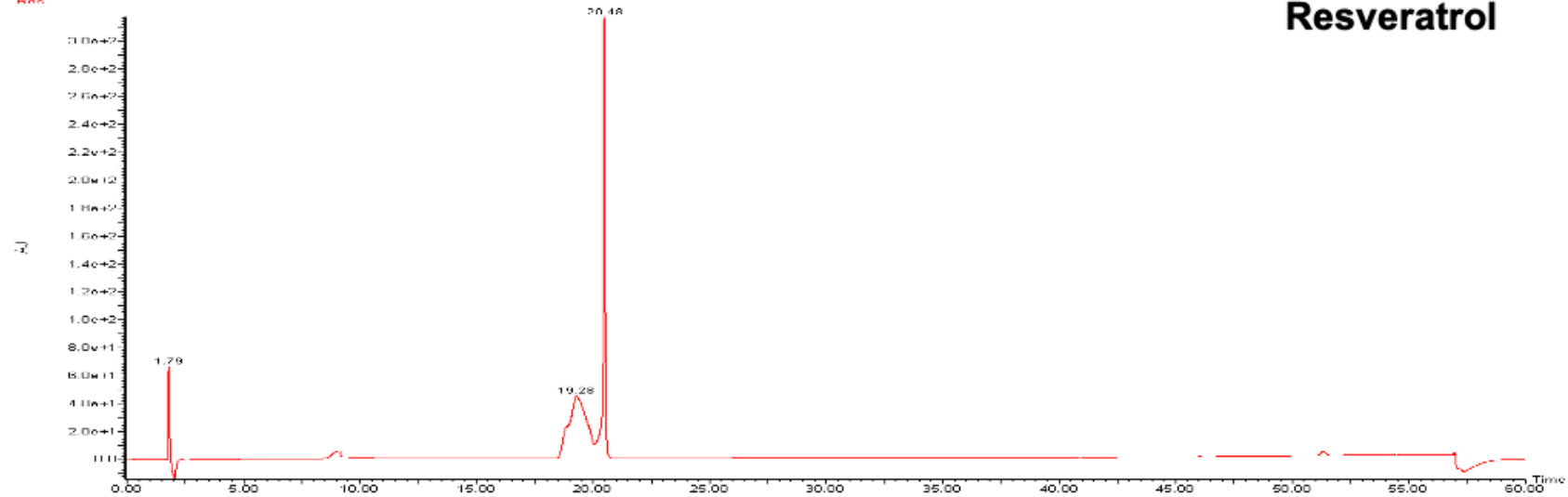

28-Jan-2016 00:55:45  
Gr

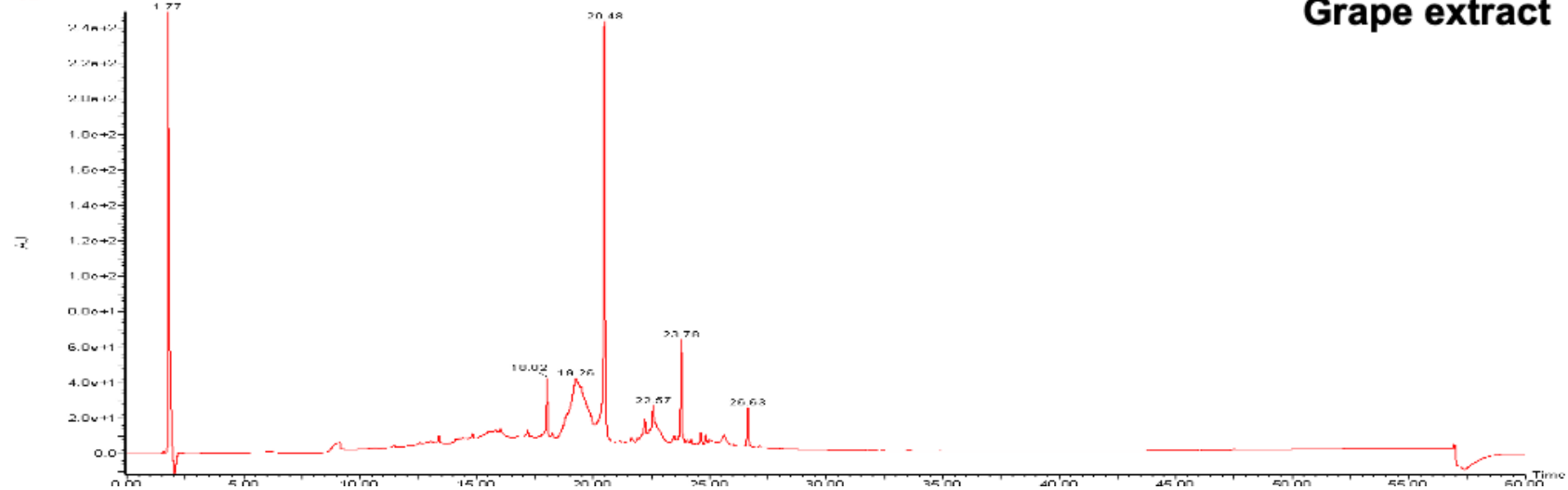

**Figure S3: HPLC analysis for resveratrol and grape extract**

Supplement: Supplementary file 1 [file molecules-26-05959-s001.zip › Molecules-Supplemental FigureS3.pdf]

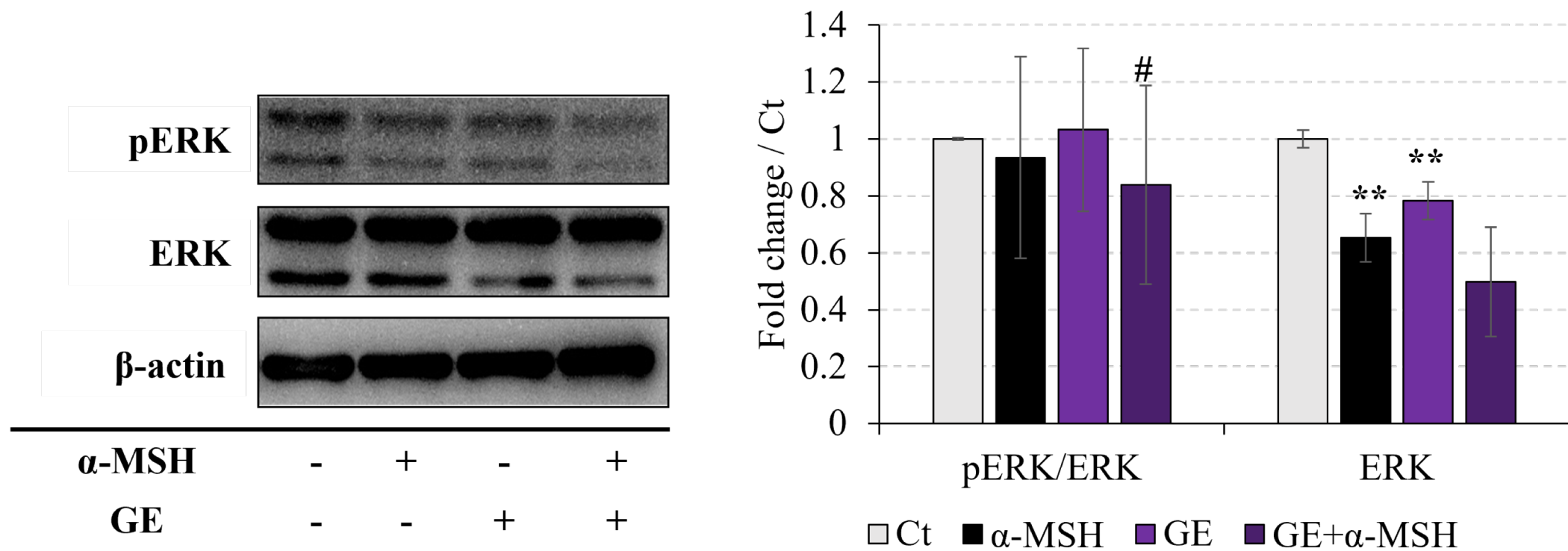

**Figure S2:** Grape extract decreased the relative phosphorylation level of Erk.

Supplement: Supplementary file 1 [file molecules-26-05959-s001.zip › Molesules-Supplemental FigureS2.pdf]
